# Stripe rust resistance gene* Yr34* (synonym *Yr48*) is located within a distal translocation of *Triticum monococcum* chromosome 5A^m^L into common wheat

**DOI:** 10.1007/s00122-021-03816-z

**Published:** 2021-03-31

**Authors:** Shisheng Chen, Joshua Hegarty, Tao Shen, Lei Hua, Hongna Li, Jing Luo, Hongyu Li, Shengsheng Bai, Chaozhong Zhang, Jorge Dubcovsky

**Affiliations:** 1 Peking University Institute of Advanced Agricultural Sciences, Weifang, 261000 Shandong China; 2 Department of Plant Sciences, University of California, Davis, CA95616 USA; 3 Howard Hughes Medical Institute, Chevy Chase, MD 20815 USA

## Abstract

***Key message*** **The stripe rust resistance gene** ***Yr34 *was transferred to polyploid wheat chromosome 5AL from* T. monococcum*** **and has been used for over two centuries.**

Wheat stripe (or yellow) rust, caused by *Puccinia striiformis* f. sp. *tritici* (*Pst*), is currently among the most damaging fungal diseases of wheat worldwide. In this study, we report that the stripe rust resistance gene *Yr34* (synonym *Yr48*) is located within a distal segment of the cultivated *Triticum monococcum* subsp. *monococcum* chromosome 5A^m^L translocated to chromosome 5AL in polyploid wheat. The diploid wheat species *Triticum monococcum* (genome A^m^A^m^) is closely related to *T*. *urartu* (donor of the A genome to polyploid wheat) and has good levels of resistance against the stripe rust pathogen. When present in hexaploid wheat, the *T. monococcum Yr34* resistance gene confers a moderate level of resistance against virulent *Pst* races present in California and the virulent Chinese race CYR34. In a survey of 1,442 common wheat genotypes, we identified 5A^m^L translocations of fourteen different lengths in 17.5% of the accessions, with higher frequencies in Europe than in other continents. The old European wheat variety “Mediterranean” was identified as a putative source of this translocation, suggesting that *Yr34* has been used for over 200 years. Finally, we designed diagnostic CAPS and sequenced-based markers that will be useful to accelerate the deployment of *Yr34* in wheat breeding programs to improve resistance to this devastating pathogen.

**Supplementary Information:**

The online version contains supplementary material available at 10.1007/s00122-021-03816-z.

## Introduction

Wheat is a major staple food crop and provides about 20% of calories and proteins for the human population. Although over 750 million tons of wheat are harvested annually from approximately 220 million hectares globally (FAOSTAT), further increases in wheat production are needed to feed a growing human population. One way to increase wheat productivity is to reduce yield losses due to pathogens. *Puccinia striiformis* f. sp. *tritici* (*Pst*), the causal agent of wheat stripe rust (or yellow rust), is currently one of the most devastating fungal diseases threatening global wheat production. This pathogen became an increasing problem after the year 2000, when more virulent and aggressive strains of *Pst* with increased tolerance to higher temperatures emerged and spread throughout the world (Chen 2005; Hovmøller et al. 2015; Milus et al. 2009).

While effective fungicides against *Pst* are available, they are expensive and pose some health and environmental risks if not properly used. The deployment of resistance genes remains the most practical and sustainable approach to control this disease. So far, over 80 stripe rust resistance genes (*Yr1*–*Yr83*) have received official designations (Li et al. 2020), but most of them are not effective against the virulent post-2000 *Pst* races. Therefore, the search for new sources of resistance and the development of molecular markers for the effective deployment of these resistance genes is a valuable research objective.

Stripe rust resistance genes *Yr34* and *Yr48*, discovered in hexaploid wheat lines WAWHT2046 in Australia and PI 610750 in the USA, respectively, have been shown to confer partial adult plant resistance against these post-2000 *Pst* races. *Yr34* was initially mapped on the long arm of chromosome 5A, 12.2 cM distal to the awn inhibitor locus *B1* (Bariana et al. 2006). *Yr48* was also mapped to chromosome 5AL, but based on their different positions relative to the common marker *gwm291* it was initially concluded that *Yr34* and *Yr48* were different genes (Lowe et al. 2011). However, a more recent study of *Yr34* identified an error in the original map, and re-mapped this gene to the same chromosome region as *Yr48*. A large allelism test (600 F_2_ plants) failed to detect variation for the *Pst* response, suggesting that the two genes are either allelic or are tightly linked. Since both *Yr34* and *Yr48* conferred similar seedling responses to pre-2000 and post-2000 *Pst* races, it was concluded that they are the same gene, which was designated as *Yr34* based on the priority of this name (Qureshi et al. 2018).

Both the *Yr34* and *Yr48* mapping populations showed suppression of recombination in the distal region of chromosome 5AL (Lan et al. 2017; Lowe et al. 2011; Qureshi et al. 2018), which is characteristic of alien introgressions, but that can also be caused by inverted chromosome segments. In addition, the *Yr48* chromosome region showed a slight segregation distortion favoring the markers linked to the resistance allele (67% vs expected 50%) (Lan et al. 2017). Although segregation distortion can occur in both alien segments and segments from the same species, they are particularly frequent in the former. Examples of segregation distortion of alien introgressions carrying resistance genes include *Lr53*/*Yr35* in *Triticum dicoccoides* (Marais et al. 2005b), *Lr54*/*Yr37* in *Aegilops kotschyi* (Marais et al. 2005a), *Lr19* in *Agropyron elongatum* (Prins and Marais 1999) and *QYrtb.pau-5A* in *Triticum monococcum* (Chhuneja et al. 2008). Based on these observations, we hypothesized that *Yr34* may be located within an alien introgression.

To characterize the 5AL distal region carrying the *Yr34* and *Yr48* genes and the presence of a potential alien introgression, we took advantage of a previously developed wheat exome capture platform (Krasileva et al. 2017) and the recent releases of reference genome sequences for multiple *Triticum aestivum* varieties (Appels et al. 2018; Walkowiak et al. 2020).

The objectives of this study were to test the hypothesis that the lack of recombination in the distal region of chromosome 5AL including *Yr34* was the result of a chromosome translocation from a wheat relative and to characterize the distribution of this translocation in the wheat germplasm. We also aimed to identify some historic recombination events that reduced the *Yr34* introgressed region to minimize linkage drag, and to develop molecular markers to facilitate the deployment of this resistance gene in wheat breeding programs*.*

## Materials and methods

### Plant materials

As a source of *Yr48*, we used wheat accession PI 610750, which is a synthetic hexaploid wheat developed by the International Maize and Wheat Improvement Center (CIMMYT) in Mexico. Since this accession has multiple *Pst* resistance genes, we selected RIL143 from the cross UC1110 × PI 610750 that carries only the 5AL resistance gene (Lowe et al. 2011). A population of 46 F_2_ plants from the cross RIL143 × Avocet-S was used to confirm the linkage of *Yr48* with the resistance to Chinese *Pst* race CYR34. As a source of *Yr34*, we used the advanced breeding line WAWHT2046 from Australia, that expressed good level of resistance to the Australian 134 E16A + *Pst* pathotype (Bariana et al. 2006). We also included the common wheat variety Mediterranean (CItr 11587, CItr 3332 and CItr 5303) that was present in many of the pedigrees identified as carriers of the 5A^m^L introgression.

For *T. monococcum*, we generated exome capture data for lines DV92 and G3116, which were the parental lines used in the construction of the first genetic map for this species (Dubcovsky et al. 1996). For the exome capture, we used the NimbleGen assay described in Krasileva et al. (2017) and we deposited the data in the T3/Wheat database (https://triticeaetoolbox.org/wheat/). In addition, we obtained another 31 *T. monococcum* accessions from the US Department of Agriculture National Small Grains Collection (USDA-NSGC, https://npgsweb.ars-grin.gov/gringlobal/search) that were used to trace the origin of the *T. monococcum* chromosome segment introgressed into bread wheat.

### Stripe rust assays

*Yr34* and *Yr48* have remained effective to all of the virulent *Pst* isolates present in California since first tested in 2009. To test if *Yr34* and *Yr48* confer resistance to Chinese *Pst* races, plants carring *Yr34* or *Yr48* and F_2_ individuals from the cross RIL143 × Avocet-S were challenged with the virulent *Pst* race CYR34 identified in 2008 (also named V26) (Liu et al. 2010). WAWHT2046, RIL143, Avocet-S and F_2_ plants from the mapping population were grown in controlled walk-in growth chambers at 24 °C during the day and 22 °C during the night. At the jointing stage, plants were inoculated with fresh urediniospores of race CYR34 mixed with talcum powder at a 1:20 ratio using the shaking off method (Ma et al. 2016; Wang et al. 2020b). Wheat leaves were uniformly dusted with this mixture of urediniospores and talc. The inoculated plants were kept in a dark dew chamber set at 10 °C for ~ 24 h and then moved back to the same walk-in growth chamber set at 18 °C during the day and 15 °C during the night. Infection types were recorded ~ 20 days after inoculation using a 0–4 scale (Liu 1988). For each wheat accession, five fully infected leaves were photographed. Sporulation area was calculated using the image analysis software ASSESS version 2.0 from the American Phytopathology Society as reported previously (Lamari 2008).

### Sequences and SNPs

Single nucleotide polymorphisms (SNPs) of *T. monococcum* accessions DV92 and G3116 were obtained from exome capture data. Exome-capture data of tetraploid wheat accessions Zavitan, D447-DW1, Kronos, Svevo, Gredho, and 280–1-*Yr15*, and hexaploid wheat accessions PI 610750 (CAP2), Billings, Inayama, Altamo, LCD_Star, PI 70613, CO960293, W7984, Berkut, MN98550-5, McNeal, CItr 7635, UC1036, Dayn, Platte, SS_MVP57, UI_Platinum, C0940610, Overley, RioBlanco, Cheyenne, Duster, Hank, 16REG01643, TAM112, TAM111, RAC875, SY_Capstone, Dharwar_Dry, IDO444, 16REG01644, 26R61, Lyman, Reeder, Opata, Excalibur, LA95135, AGS2000, RSI5, Choteau, 2045A, Vida, Bakahtawar94, CCW3A37, KS05HW14-3, CCW3A49, MN99394-1, PBW343 and UC1419, were obtained from the T3/Wheat database (https://triticeaetoolbox.org/wheat/). The published reference genomes of hexaploid wheat Chinese Spring (Appels et al. 2018) and another 10 wheat varieties in the Wheat Pan Genome project (Walkowiak et al. 2020) were included in our analyses. The SNPs identified by exome capture data from two different wheat panels were used to study the distribution of the *Yr34* translocation. The first wheat panel consists of 982 hexaploid genotypes (http://wheatgenomics.plantpath.ksu.edu/1000EC/) (He et al. 2019). The second panel includes 460 hexaploid accessions (Pont et al. 2019). A total of 1,442 hexaploid wheat genotypes were obained with exome sequencing data, and they were separated in four historical groups (every 30 years): group I, before 1930; group II, from 1931 to 1960; group III, from 1961 to 1990; and group IV, after 1991.

### Marker development and SNP validation

Genome-specific primers were designed with software Primer3 (https://bioinfo.ut.ee/primer3-0.4.0/primer3/) to amplify gene regions carrying putative *T. monococcum*-specific SNPs. Techniques and procedures for developing cleaved amplified polymorphic sequence (CAPS) markers were reported previously (Konieczny and Ausubel 1993). NEBcutter V2.0 (http://www.labtools.us/nebcutter-v2-0/) was used to detect restriction sites including the targeted SNPs. PCR reactions were performed in a Veriti 96-Well Fast Thermal Cycler (Applied Biosystems) with an initial denaturation step of 94 °C for 5 min, followed by 40 cycles of 94 °C for 30 s, 50–65 °C for 30 s, and 72 °C for 1 min, with a final extension step at 72 °C for 7 min. The PCR products were visualized in 1–2.5% agarose gels stained with ethidium bromide. The PCR amplification products with the right sizes were then sequenced to confirm the present of 5A^m^L-specific SNPs. Restriction enzymes from New England BioLabs Inc. were used to digest the amplified products.

Eleven pairs of genome-specific primers, including *pku5410F3R3*, *pku5414F4R4*, *pku5429F2R2*, *pku5451F5R5*, *pku5473F2R2*, *pku5488F4R4*, *pku5497F2R2*, *pku5507F1R1*, *pku5508F1R1*, *pku5575F5R5* and *pku5585F1R1* (Table 1), were developed from 11 different genes distributed along the translocated *T. monococcum* segment in RIL143 to detect polymorphic sites among 32 cultivated *T. monococcum* accessions.

**Table 1:** Primers used in the present study

Markers	Forward primer	Reverse primer	Size (bp)	T (℃)	Enzyme
*pku5380F3R3*	GTACTCCGGTCAAAGTCAGTAGAGT	CTTGGCGAGCTTGCCGAAGG	741	55	
*pku5381F2R2*	TTAGCTATTCACTCTTCCGACG	TTCGTTTGATGTATGAACCCAG	1648	57	
*pku5409F1R1*	TGACTCTATAACCATCACCA	ATTTTAGCCTCTCCCTTTT	1323	50	
*pku5409F2R2*	ACTAGGCTTGCAGCTTAGAG	TTCAATGTGCGTGACACTT	1619	51	
*pku5410F3R3*	ATTCGATGCTCCATTGGTCTCC	TTGTTCTGTCCTTGCGTGCTG	1004	56	
*pku5414F4R4*	GCGTTCTGGTATCAGTTCCTTCTTAAG	TCTGGCTGTTGCCTGGTTGG	677	54	*Xho*I
*pku5429F2R2*	ATGCAAGCTATAGTTGTATCAACC	GCACCACCTATGTCACAGTTTC	779	53	*Pvu*II
*pku5451F5R5*	GGAGGTGGGCTAGGACCCTT	ACCACAGTTGTAACTTGCACGACA	1042	53	
*pku5466F2R2*	CATGGTGAGTCCTGATCGTTCG	GCCACATCCTCCTGAGCAAAA	456	57	*EcoR*I
*pku5473F2R2*	GCGCACCACCAGGTTATATC	ATAGGGTTGTGCTACTGCTAATGTA	1013	54	*BsiHKA*I
*pku5477F3R3*	TTGCCACCAAGGGACGCCA	CCACCTACTACCAGGGACCCGATT	607	60	
*pku5488F4R4*	TGTGATAGTTCTAGTATGTAGTAACCG	CCGTGTCAGCACATTCTCAAC	1079	52	*Apo*I
*pkuS5A7761F2R2*	CTCTCCGTTGGTGTCAGCCACTTA	CATCATGCCGACCGCATTCC	504	60	
*pku5492F1R1*	AAAGCCCTGCTCAAGACT	TTATTTTCGGGATCCAAC	629	55	*Mse*I
*pku5494F1R1*	ACGATCACTATGGTTAATGATCATG	GGAGGGAAGGTAGGAAGGCAT	842	57	
*pku5497F2R2*	GATGGCAGTCTTGTGGTATG	AAATAGATCTGGGCAATGTATC	1188	51	*Pvu*II
*pku5507F1R1*	CAAGTAGCTAGCAGCAAGCAAAGATC	GCGTTCACGCACGCAGTCA	884	60	*HpyCH4*IV
*pku5508F1R1*	GTCGTCCATATTGGTTTTGGTG	GGTGTAAACGCACTTGCTTGG	1219	52	*Hinf*I
*pku5515F2R2*	TTGCACTGCTTTTTCTTCATT	CAACACATACGCCCATCTCT	1201	58	
*pku5525F1R1*	GCAGCCGCCTCGTCTGATGA	GGATGGCGTGAGCCTTGAGC	873	61	
*pku5527F1R1*	GCAAAGCACTTCCGATGA	TTGTACCTCCCGAGCAGT	1158	55	
*pku5542F1R1*	CTGGCCGTGACAGATGTAGACT	TTCATTTCGGTGTTGGTTTGC	332	53	*Hpy188*III
*pku5559F4R4*	CCACATAAACGTAATGTTCACACTG	AATTTCGACCTACTTTCCCTA	1005	51	
*pku5575F3R3*	ATTAGATGGATTGCCAGGA	TGTTTAAGTACACAAAGAAGCTCT	950	52	
*pku5575F5R5*	CTGCAACTCATTCTATTTGGT	GTTTAGTTCCAGGTGCTTCA	881	53	*Acu*I
*pku5585F1R1*	GCTAGGTATTGGAGGATCCGAAT	AATATAAGTACCAATGCCCTACCAA	1367	55	*Ban*II
*pkuAri5827F3R3*	GTTACTTTCCACCGTGAGCG	CGTCTGAGCCAAACAAGGATT	1021	55	
*Vrn2F3R3*	GATCACAATTATTGATGAAGTTTCG	TCACTAACCGACTTATTTTACTTTG	761	56	
*Vrn2F5R5*	TCCTCGCTGGTTCTCATTCAAG	TCCATTCCCGCATATCTTTT	1108	55	
*Zcct2F6R6*	GAGCGATTGAGTTTGGACTGTGCG	GGCGAAGCTGGGGGTGACGA	515	58	
*R3C1N3/RACEC1N1*	GCAATCATGACTATTGACACA	GGGCGAAGCTGGAGATGATG	230	55	

### Candidate genes and expression analysis

Candidate genes for our target region were identified from the genome sequences of two European winter wheat varieties Arina*LrFor* and SY Mattis included within the 10 + Wheat Genomes Project, and that were the most similar to the lines carrying *Yr34* in the distal region of chromosome 5AL. Sequences were obtained from the database of the Institute of Plant Genetics and Crop Plant Research (IPK) (https://webblast.ipk-gatersleben.de/wheat_ten_genomes/viroblast.php). The expression levels for the candidate genes were obtained from the Wheat Expression Browser (expVIP, http://www.wheat-expression.com/) (Borrill et al. 2016).

## Results

### Assessment of stripe rust responses

**Fig. 1:**
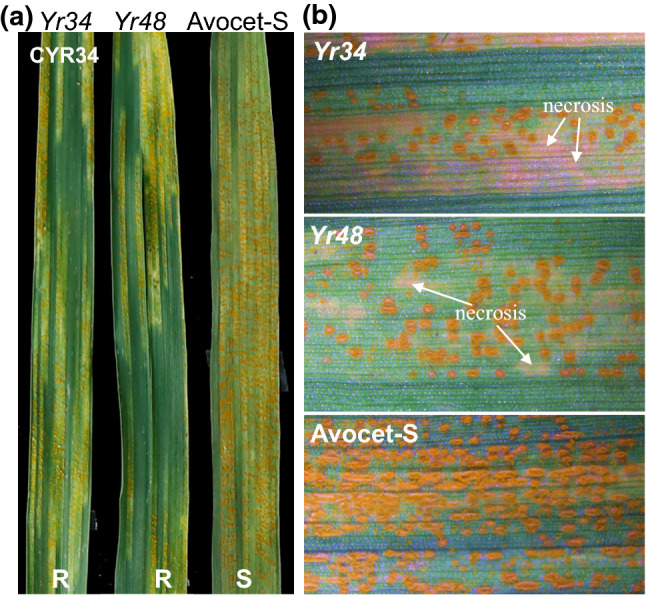
Stripe rust reactions to *Pst* race CYR34 inoculated on leaves of WAWHT2046 (*Yr34*), RIL143 (*Yr48*) and Avocet-S (susceptible control). Infected leaves visualized by **a** a digital camera and **b** a microscope (Zeiss Discovery V20) at the adult-plant stage. Necrotic responses are marked with arrows

WAWHT2046 and RIL143 exhibited a moderate level of resistance to the virulent Chinese race CYR34, whereas the control line Avocet-S was susceptible (Fig. 1a). To quantify the amount of disease present on the leaves, disease measurement were performed on five fully infected leaves of each line using software ASSESS v2. The average percentage of the leaf area covered by *Pst* pustules was significantly lower (*P* < 0.001) in WAWHT2046 (24%, ranging from 15 to 34%) and RIL143 (29%, ranging from 18 to 39%) than in Avocet-S (84%, ranging from 72 to 95%). Chlorotic/necrotic responses are marked with arrows on the leaves of WAWHT2046 and RIL143 in Fig. 1b.

Since RIL143 carries only one of the resistant alleles (*Yr48*) segregating in the UC1110 × PI 610750 RIL population, we hypothesized that its resistance to *Pst* race CYR34 was conferred by this allele*.* This was confirmed by phenotyping 46 F_2_ plants derived from the cross RIL143 × Avocet-S with CYR34, and genotyping them with marker *cfa2149*, which is completely linked to *Yr48* (Lowe et al. 2011). All ten plants homozygous for the RIL143 allele were resistant, whereas all 12 plants homozygous for the Avocet-S allele were susceptible, confirming that RIL143 resistance to race CYR34 is linked to the *Yr48* region.

### *T. monococcum* segments introgressed into polyploid wheat

To explore the origin of the *Yr34* segment, we compared the SNPs from the exome capture of *Yr48* donor line PI 610750 with that of 48 other hexaploid wheat accessions, 6 tetraploid lines, and two diploid *T. monococcum* lines (DV92 and G3116) generated as part of the USDA-NIFA funded WheatCAP project. This SNP dataset is available in the T3/Wheat database (https://triticeaetoolbox.org/wheat/genotyping/display_genotype.php?trial_code=2017_WheatCAP_UCD). To our surprise, we found that the distal region of chromosome arm 5AL in PI 610750 had a large number of rare polymorphisms that were shared with *T. monococcum* accessions DV92 and G3116 and the common wheat variety Billings. These results suggested, for the first time, that the distal region of 5AL carrying *Yr34* could have originated in *T. monococcum.* To explore this region in more detail, we focused on the SNPs that were present in the two *T. monococcum* accessions, but were absent in all other accessions of polyploid wheat (except PI 610750 and Billings), and that are referred hereafter as *T. monococcum-*specific SNPs.

**Fig. 2:**
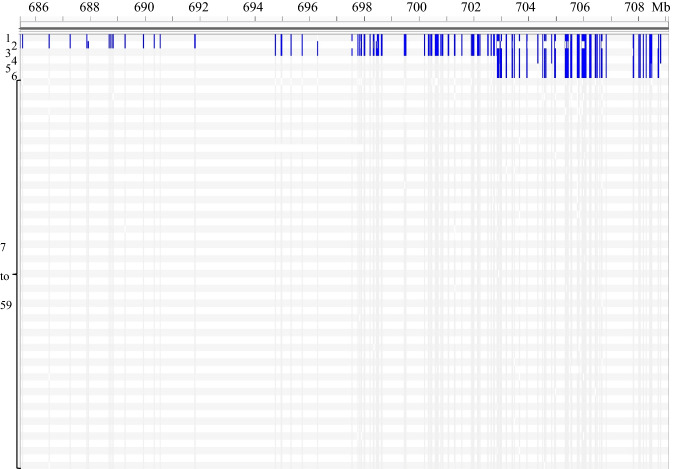
Distribution of 1,047 *T. monococcum*-specific SNPs on the distal 24.3 Mb of chromosome 5AL (685.4 Mb to end). 1, DV92; 2, G3116; 3, PI 610,750 (*Yr48*); 4, Billings; 5, Arina*LrFor*; 6, SY Mattis; 7–59, tetraploid and hexaploid wheat accessions from exome-capture sequencing (Table S1). This figure was produced using the Integrative Genomics Viewer (IGV) software version 2.8.9 (Robinson et al. 2011). Vertical lines in blue represent *T. monococcum*-specific SNPs whereas lines in light grey are normal wheat SNPs or not-polymorphic sites with Chinese Spring. Coordinates were based on CS RefSeq v1.0

In the 5AL region starting from 685.4 Mb to the end of the chromosome (based on CS RefSeq v1.0 coordinates), we identified 1,047 *T. monococcum*-specific SNPs (Table S1). To visualize the distribution of these SNPs in the distal 24.3 Mb of chromosome arm 5AL, we represented the *T. monococcum*-specific SNPs in blue and other SNPs in grey (Fig. 2). This figure shows that the *T. monococcum* segment in PI 610750 was approximately 15 Mb long, extending from 694.8 Mb to the end of the chromosome and sharing 1,019 *T. monococcum*-specific SNPs with DV92 and G3116. The *T. monococcum* segment in Billings was approximately 8 Mb shorter (~ 7 Mb long), and extended from 702.9 Mb to the end of the chromosome sharing 569 *T. monococcum*-specific SNPs with DV92 and G3116 (Fig. 2).

To test if the *T. monococcum* translocation was present in other sequenced *T. aestivum* accessions, we performed BLASTN searches using the sequences flanking the target SNPs. We found that only the two European winter wheat varieties ‘Arina*LrFor*’ and ‘SY Mattis’ have the distal 5AL *T. monococcum* translocation among the ten *T. aestivum* accessions assembled as pseudomolecules in the Wheat Pan Genome project (Walkowiak et al. 2020). These two varieties share the 569 *T. monococcum*-specific SNPs identified in Billings (Table S1) indicating that they have the same translocated segment. Using the genomic sequences of Arina*LrFor* and SY Mattis, we were able to estimate more precisely the size of the *T. monococcum* introgression in these two varieties, which was approximately 9.5 Mb, and extended from 700.7 Mb to the end of the chromosome (710.1 Mb) in Arina*LrFor* and from 693.1 Mb to the end of the chromosome (702.6 Mb) in SY Mattis.

**Fig. 3:**
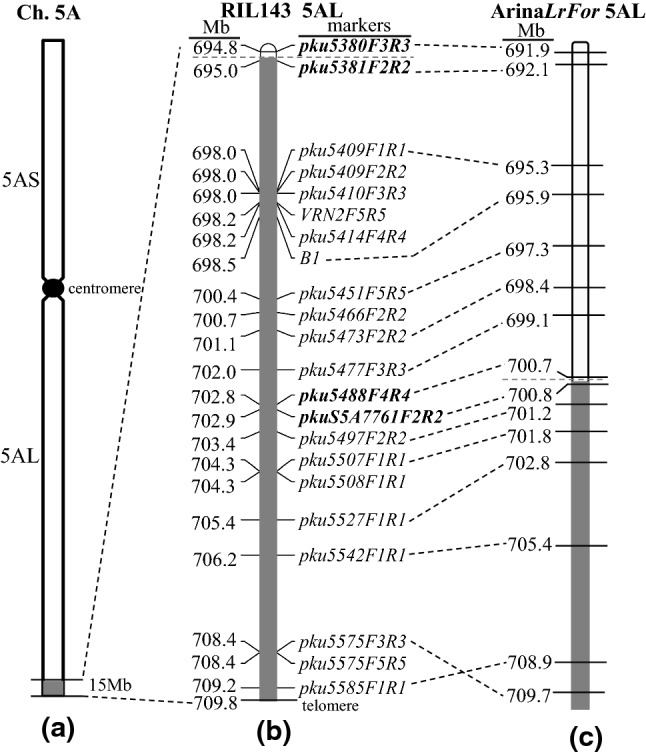
*T. monococcum* chromosome segments introgressed into common wheat. **a** Chromosome 5A; **b** The length of the introgressed *T. monococcum* segment in RIL143 (grey) is estimated to be 15 Mb based on CS coordinates, but can be adjusted to 17.5 Mb based on Arina*LrFor* coordinates; **c** The introgressed *T. monococcum* segment in Arina*LrFor* (grey) was originally estimated to be 7 Mb based on CS coordinates and was adjusted to 9.5 Mb based on Arina*LrFor* coordinates. Coordinates in (**b**) and (**c**) are based on CS RefSeq v1.0 and Arina*LrFor*, respectively. The dotted horizontal grey lines indicate the recombination events between *T. monococcum* and *T. aestivum* chromosomes

Since the *T*. *monococcum* segment in PI 610750 was approximately 8 Mb longer than in Arina*LrFor* and SY Mattis (Fig. 2), we adjusted the estimate of its length from 15 Mb to 17.5 Mb (distal 9.5 Mb in Arina*LrFor* + proximal 8 Mb estimate based on Fig. 2). To define better the translocation breakpoint in PI 610750, we developed 21 A/A^m^-genome specific primers across the 17.5 Mb introgressed *T. monococcum* chromosome segment (Table 1) and used them to test DV92, RIL143, Arina*LrFor* and WAWHT2046 via Sanger sequencing (Table S2). The physical positions of these markers are presented in Fig. 3. Using these markers, we determined that the translocation point in RIL143 occurred between markers *pku5380F3R3* and *pku5381F2R2* (Fig. 3b, 694.8 and 695.0 Mb in CS, respectively). Using additional SNP polymorphisms, we determined that the border of the translocation in Billings, Arina*LrFor* and SY Mattis was between markers *pku5488F4R4* and *pkuS5A7761F2R2* (Fig. 3c, 702.8 and 702.9 Mb in CS, 700.7 and 700.8 Mb in Arina*LrFor*).

**Fig. 4:**
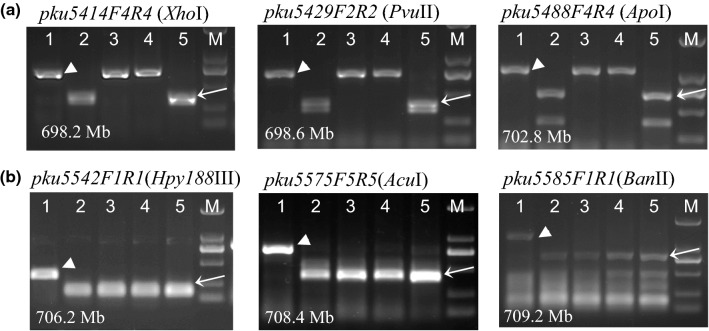
CAPS markers used to characterize the *T. monococcum* segment present in WAWHT2046 (*Yr34*). **a** CAPS markers *pku5414F4R4* (698.2 Mb, *Xho*I), *pku5429F2R2* (698.6 Mb, *Pvu*II) and *pku5488F4R4* (702.8 Mb, *Apo*I) in the region that differentiates RIL143 and Arina*LrFor*; **b** CAPS markers *pku5542F1R1* (706.2 Mb, *Hpy188*III), *pku5575F5R5* (708.4 Mb, *Acu*I) and *pku5585F1R1* (709.2 Mb, *Ban*II) in the common *T. monococcum* segment. 1, Avocet-S (wheat control); 2, RIL143 (*Yr48*, long introgression control); 3, WAWHT2046 (*Yr34*); 4, Arina*LrFor* (short introgression control); 5, DV92 (*T. monococcum* control); M, markers. Coordinates are based on CS RefSeq v1.0. Arrowheads represent wheat bands and arrows represent *T. monococcum* bands

We then explored the presence of the *T. monococcum* translocation in WAWHT2046, which is the original line where *Yr34* was discovered. PCR markers in the region that differentiates RIL143 and Arina*LrFor* (*pku5414F4R4*: 698.2 Mb, *pku5429F2R2*: 698.6 Mb and *pku5488F4R4*: 702.8 Mb, CS RefSeq v.1 coordinates) showed the *T. monococcum* allele in RIL143 and DV92 but not in WAWHT2046, Arina*LrFor* or the wheat control Avocet-S (Fig. 4a). By contrast, PCR markers in the common *T. monococcum* distal segment (*pku5542F1R1*: 706.2 Mb, *pku5575F5R5*: 708.4 Mb and *pku5585F1R1*: 709.2 Mb) showed the *T. monococcum* allele in RIL143, WAWHT2046, Arina*LrFor* and DV92, but not in Avocet-S (Fig. 4b). These results confirmed that WAWHT2046 carries the same *T. monococcum* translocation as Arina*LrFor*. We further confirmed that the borders of the translocation were identical using flanking markers *pku5488F4R4* and *pkuS5A7761F2R2* described above, and that all the tested SNPs starting from position 702.9 Mb were identical in Arina*LrFor* and WAWHT2046 (Table S2).

### Distribution of *T. monococcum* introgressions in hexaploid wheat

In order to determine the frequency and the distribution of the *T. monococcum* translocated segments in wheat genotypes, 1,442 hexaploid wheat accessions with exome sequencing data derived from the 1,000 wheat exomes project (includes 982 hexaploid wheat genotypes) (He et al. 2019) and the 500 exomes project (includes 460 hexaploid wheat genotypes) (Pont et al. 2019), were used for SNP calling and comparative analysis. Our survey revealed that 105 accessions out of 982 (10.7%) in the first panel and 147 accessions out of 460 (32%) in the second panel possess *T. monococcum*-wheat translocations on the distal region of chromosome 5AL (Table S3 and S4). Among these 252 accessions plus the 5 lines mentioned before carrying the *T. monococcum*-wheat translocation (PI 610750, Billings, Arina*LrFor*, SY Mattis and WAWHT2046), we identified *T. monococcum* segments of 14 different lengths, which were designated as L1 to L14 (Table S3 and S4).

**Fig. 5:**
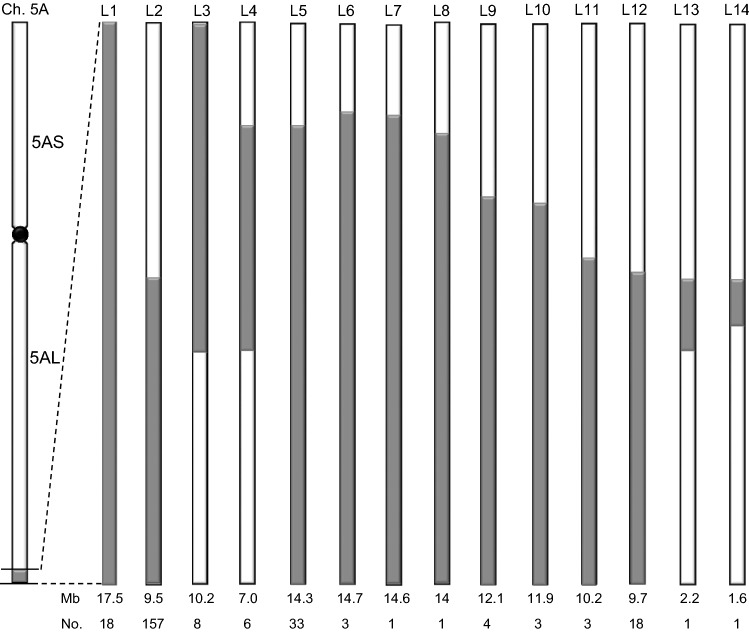
Estimated lengths of the L1-L14 *T. monococcum* introgressions. L1 is the longest (and likely original translocation) and all others were generated by distal or proximal recombination events with chromosome arm 5AL. The first row of numbers below the figure indicates the lengths of the *T. monococcum* segment estimated using a combination of coordinates of the 9.5 Mb *T. monococcum* segment in the Arina*LrFor* genome and the CS RefSeq v1.0 coordinates for the rest of the *T. monococcum* segment not present in Arina*LrFor*. The second row of numbers indicated the number of wheat accessions carrying each introgression. The *T. monococcum* introgression is indicated in gray and the *T. aestivum* segment in white

The length of the introgressed *T. monococcum* segments and the number of lines carrying each introgression are indicated at the bottom of Fig. 5, and a complete list of accessions is available in Table S5. Lines carrying translocations L5 to L11 include the complete L2 region present in WAWHT2046 and, therefore, are likely carriers of the *Yr34* resistance gene. By contrast, translocations L3, L4, L13 and L14 include only part of the L2 translocation, so we currently do not know if they carry the *Yr34* resistance gene.

Using the data from the two exome capture projects (He et al. 2019; Pont et al. 2019), we were able to find a small amount of SNPs among the different introgressions. Since most of these SNPs were frequent in the *T. aestivum* accessions without the *T. monococcum* introgressions, we interpreted them as conversion events. Supplemental Table S6 summarizes the 11 most likely conversion events based on the presence of at least two adjacent SNPs separated by less than 4 kb and all identical to *T. aestivum* alleles. The raw SNPs data used in this analysis are presented in Tables S3 and S4, which also include 23 additional single SNPs. These single SNPs were all frequent in hexaploid lines without the *T. monococcum* introgressions, and could also be conversion events (not included in Table S6).

The previous results suggest that the *T. monococcum* introgressions of different lengths may have originated by recombination events from a single *T. monococcum* introgression. This hypothesis is also supported by shared borders among several of the accessions. For example, the L3 introgression shared its proximal border with L1 (between SNPs *S5A_694759680* and *S5A_694966923*, Table S3). This was further confirmed by a more precise mapping of the L1 and L3 proximal border to a 0.2 Mb region between markers *pku5380F3R3* and *pku5381F2R2* in accessions PI 619381 and PI 619379. These results suggest that L3 likely originated from L1 by a distal recombination event with 5AL. This distal border of L3 is shared by L4, which also shares a proximal border with L5 suggesting a possible origin of L4 from recombination between L3 and L5. Similarly, the L13 introgression shares the proximal border with L2 and the distal border with L3 and L4, so it could have originated from recombination between L2 and either L3 or L4. L14 shares the proximal border with L13 and could have originated by a distal recombination event with 5AL in L13. More precise mapping of the shared borders will be required to validate these hypotheses. Finally, all other *T. monococcum* introgressions share the most distal *T. monococcum* SNPs and are likely terminal introgressions derived by proximal recombination events between 5AL and L1 or other lines with longer distal *T. monococcum* introgressions.

We detected the 5A^m^L translocations of different lengths in 50 countries covering all continents where wheat is grown (Table S7), especially in European countries, suggesting a wide distribution. The overall frequency of the 5A^m^L translocation segments in the present panel of hexaploid wheat genotypes was 17.5% (252/1442), but the proportion in different continents varied significantly (Fig. S1 and Table S7). More specifically, the translocation was detected in 34.4% wheat accessions from Europe, 8.1% accessions from North America, 8.8% accessions from Asia, 2.9% accessions from Oceania, 4.8% accessions from Africa, and 2.1% accessions from South America (Fig. S2 and Table S7).

We then compared the frequencies of the translocation within four historical groups (every 30 years). In the first wheat panel (He et al. 2019), we found that the translocation was rare (1.6%) in varieties released before 1930 (Group I), but its frequency increased sharply (57.1%) in the modern varieties released after 1990 (Group IV). Likewise, in the second wheat panel (Pont et al. 2019), the frequency of the translocation increased from 11.1% in Group I to 46.3% in the more recent varieties of Group IV. In summary, this analysis revealed rapid increases in the frequency of 5A^m^L.5AL translocation in hexaploid wheat varieties (Table S8).

### Tracing the origin of the RIL143/Billings translocation

To trace the origin of the translocation, we performed pedigree analysis to determinate the relationship among wheat accessions carrying the translocations using the wheat pedigree database (http://www.wheatpedigree.net/). Among the 135 lines carrying the 5A^m^L translocations for which we obtained pedigree information, we found that 103 shared a common parental line named “LV-Mediterranean” (or its derivatives) in their pedigree (Table S9). We found no information about LV-Mediterranean, but we found that its derivative “Mediterranean”, was a late-sown variety introduced into the U.S. from Europe in the year 1819 (Olmstead and Rhode 2002).

We identified three accessions of Mediterranean in the USDA-NSGC (CItr 3332, CItr 5303 and CItr 11587, received in 1912, 1913 and 1933, respectively) and characterized them with markers *pku5380F3R3* (694.8 Mb), *pku5381F2R2* (695.0 Mb), *pku5473F1R1* (701.1 Mb), *pku5497F2R2* (703.4 Mb), *pku5542F1R1* (706.2 Mb) and *pku5585F5R5* (709.2 Mb) (Table 1). These markers showed that CItr 3332 does not carry the *T. monococcum* translocation, and that both CItr 5303 and CItr 11587 carry a *T. monococcum* translocation with a common proximal border and different distal borders. The proximal border was located between 694.8 and 695.0 Mb using flanking markers *pku5380F3R3* and *pku5381F2R2*, in the same position as L1 and L3. The 5A^m^L alleles extended to 709.2 Mb for line CItr 5303, but only to 703.4 Mb for line CItr 11587, with 5AL alleles for markers between 706.2 Mb and 709.2 Mb. These results suggest that CItr 5303 carries the same L1 translocation as PI 610750, whereas CItr 11587 is likely to carry the L3 translocation (Fig. 5). These results suggest that Mediterranean (or LV-Mediterranean) could be the origin of the *T. monococcum* translocation or at least of its introduction in North America.

To further characterize the *T. monococcum* introgression in Mediterranean, we determined the alleles present in CItr 5303 for SNPs detected between the L1 translocation in PI 610750 and the L2 translocation in Arina*LrFor*. A comparison of the PI 610750 exome capture data with the genomic sequence available for Arina*LrFor* (Walkowiak et al. 2020) revealed 13 polymorphisms between L1 and L2. Six of these SNPs appear to be also the result of conversion events in PI 610750 based on the presence of the same SNPs in several of the sequenced *T. aestivum* genomes (Table S10) and their absence in Mediterranean. The other seven polymorphisms (including one 34-bp deletion and six SNPs, Table S10) were not present in any of the 10 sequenced *T. aestivum* pseudomolecules, including Arina*LrFor* and SY Mattis, nor in the variety Mediterranean (accession CItr 5303). These results suggest that these polymorphisms originated in PI 610750 after the introgression of the *T. monococcum* segment in *T. aestivum*.

### Identification of the closest source of the *T. monococcum* segment

To investigate the origin of the *T. monococcum* segment and to explore the source of the seven polymorphisms between PI 610750 and Mediterranean that we were not able to find in *T. aestivum*, we sequenced the regions including these SNPs and several other regions in a set of 32 accessions of cultivated *T. monococcum* subsp. *monococcum.*

We focused on the cultivated accessions of *T. monococcum* because a comparison of the exome capture data from the cultivated accession DV92 and the wild *T. monococcum* subsp. *aegilopoides* accession G3116, revealed that the L1 introgression shared 249 out of 301 SNPs (82.7%) with DV92 and only 52 (17.3%) with G3116 (Table S11). The numbers were similar for the 9.5-Mb distal region, where the L2 translocation shared 89.5% SNPs (137/153) with DV92 and 10.5% (16/153) with G3116 (Table S11). These results indicate that the translocated segment originated from the cultivated *T. monococcum* subsp. *monococcum*.

**Fig. 6:**
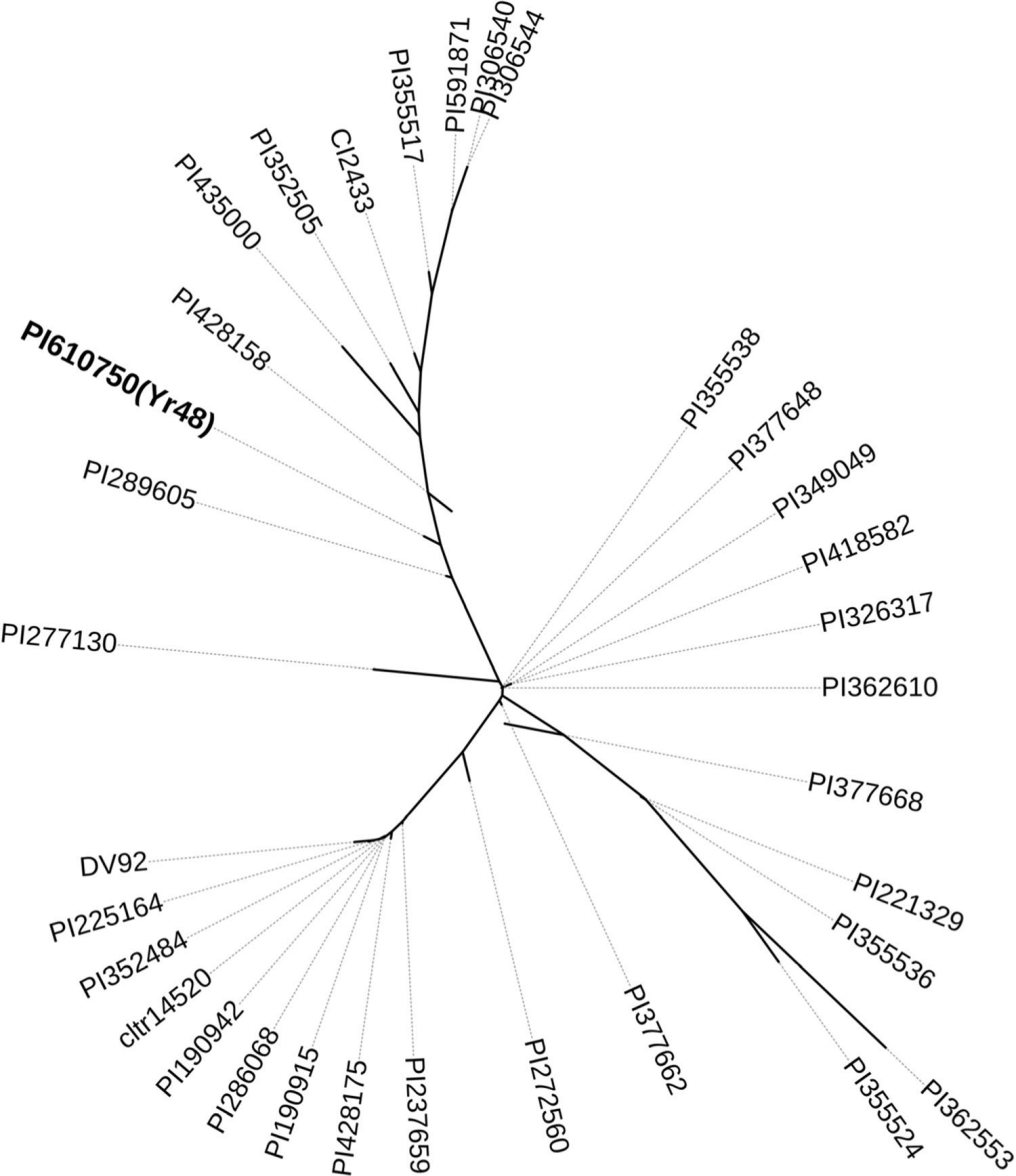
SNP-based phylogenetic analysis. Sequences were aligned with Muscle as implemented in software Mega version 7. Phylogenetic tree was produced based on 67 polymorphisms identified among 9,435 bp obtained by Sanger sequencing from 11 loci. The evolutionary history was inferred using the Neighbor-Joining method. All ambiguous positions were removed for each sequence pair (pairwise deletion option). Interactive Tree Of Life (iTOL) version 5 was used to visualize the tree (https://itol.embl.de/). PI 610750 (*Yr48*) is highlighted in bold. Putative conversion polymorphisms listed in Tables S6 and S10 that were not present in the L1 introgression in Mediterranean were excluded in the comparisons between PI 610750 and the *T. monococcum* accessions

We evaluated the relationships among 32 *T. monococcum* subsp. *monococcum* accessions by Sanger sequencing of 11 different gene regions across the L1 introgression (Table S12). Since our objective was to find the closest *T. monococcum* accession to the original translocation, we eliminated all the PI 610750 SNPs that were not in Mediterranean CItr 5303 (Table S10). We failed to detect polymorphisms among the 32 *T. monococcum* accessions and PI 610750 in the 3,300 bp amplified with primers for four regions (*pku5410F3R3*, *pku5414F4R4*, *pku5507F1R1* and *pku5508F1R1*). For the other 7 regions, we sequenced 6,135 bp that revealed 67 polymorphic sites (Table S12). A Neighbor-Joining tree based on these polymorphisms (Fig. 6) showed that PI 610750 was located within a cluster that included multiple European accessions (Table S13). The two closest *T. monococcum* accessions to PI 610750 were PI 289605 and PI 428158, which were both collected in the United Kingdom. The *T. monococcum* accession PI 289605 showed only 4 SNPs with the L1 introgression in hexaploid wheat (99.958% identical, excluding the PI 610750 unique SNPs not present in Mediterranean), suggesting that this accession is closely related to the one that was the source of the 5A^m^L.5AL translocation.

Among the seven polymorphisms present in PI 610750 and not in the *T. aestivum* without the *T. monococcum* introgression (Table S10), two (RefSeq v1.0 coordinates, 703,182,334 and 707,059,015 bp) were also absent in the 32 accessions of *T. monococcum*. This result suggests that these two SNPs may have originated either by mutations in PI 610750 or by conversion from *T. aestivum* accessions with different haplotypes than the ones included in our study. Interestingly, the other 5 polymorphisms that were not present in any of the sequenced *T. aestivum* genomes (one 34-bp deletion: 705,376,608–705,376,641 bp and SNPs 705,375,944, 705,376,462, 705,408,362 and 705,408,374 bp, Table S10) were found in a group of four *T. monococcum* accession from Armenia, Azerbaijan and Germany (PI 326317, PI 418582, PI 349049 and PI 355524, Table S13). This result suggests the intriguing possibility of recombination with a different *T. monococcum* accession, but more extensive surveys and sequencing will be required to test this hypothesis.

The vernalization locus *VRN2* is included in the *T. monococcum* introgression region present in L1 but not in L2 (CS RefSeq v1.0 coordinates, 698.2 Mb, Fig. 3b). This locus includes linked genes *ZCCT1* and *ZCCT2*, and both genes are not functional in the A genome of polyploid wheat (Distelfeld et al. 2009). The *T. monococcum VRN2* alleles for a spring growth habit have either a deletion of both *ZCCT1* and *ZCCT2* that can be identified with primers *Vrn2F3R3*, *Zcct2F6R6* and *R3C1N3/RACEC1N1* (Table 1) or non-functional copies in both genes characterized by an arginine (R) to tryptophan (W) mutation at position 35 of the CCT domain in the ZCCT1 protein (henceforth RW mutation) that can be detected with CAPS marker *R3C1N3/RACEC1N1* (Table 1) (Yan et al. 2004). Analysis of PI 610750 and Mediterranean showed that both accessions carry the *ZCCT1* and *ZCCT2* deletion. We then screened a collection of 32 cultivated *T. monococcum* accessions, enriched in the presence of the *ZCCT1* and *ZCCT2* deletion based on a previous survey (Yan et al. 2004). Analysis with *Vrn2F3R3*, *Zcct2F6R6* and *R3C1N3/RACEC1N1* identified 9 accessions where the functional *VRN2* alleles were present, 4 carrying the RW mutation and 19 carrying the deletion of both genes (Table S13). Interestingly, among the *T. monococcum* accessions within the cluster of European varieties including PI 610750, only accession PI 591871 from Georgia showed the *VRN2* deletion. Since this was not the closest accession to PI 610750, the origin of the *VRN2* deletion in the introgressed L1 segment remains an open question.

### Candidate genes for *Yr34*

*Yr34* was initially mapped to the distal region of chromosome 5AL in WAWHT2046 (Qureshi et al. 2018), and we show here that this region is included in the 9.5-Mb introgression from *T. monococcum* (L2). Based on these results, we concluded that the *Yr34* gene is located within the L2 translocation. Since Arina*LrFor* shares the same L2 translocation as WAWHT2046, we used the Arina*LrFor* genomic sequence (https://webblast.ipk-gatersleben.de/wheat_ten_genomes/viroblast.php) to obtain a list of 134 annotated genes in the candidate region (*TraesARI5A01G579500*- *TraesARI5A01G592800*, Table S14). The functional annotation of these genes using Pfam or BLASTN/BLASTX searches in GenBank did not reveal any typical NBS-LRR resistance genes but detected six genes annotated as putative *RECEPTOR-LIKE PROTEIN KINASES* (*RLKs, TraesARI5A01G582700*, *TraesARI5A01G584100*, *TraesARI5A01G586200*, *TraesARI5A01G589400*, *TraesARI5A01G591100* and *TraesARI5A01G591700*).

We then analyzed the expression levels of the candidate genes using published RNAseq studies compiled in the wheat expVIP database (http://www.wheat-expression.com/). Among the 134 genes annotated in the candidate gene region in the Arina*LrFor* genome, we found that 53 were expressed in wheat leaves infected with *Pst*, which included four of the six annotated *RLKs* genes (*TraesARI5A01G582700*, *TraesARI5A01G586200*, *TraesARI5A01G589400* and *TraesARI5A01G591700*). We have prioritized these four genes for further functional characterization.

## Discussion

### Diploid wheat *T. monococcum* is a good source of resistance genes

Diploid wheat *T. monococcum* (2*n* = 2*x* = 14, A^m^A^m^) is closely related but a different species from *T. urartu* (2*n* = 2*x* = 14 = AA) (Johnson and Dhaliwal 1976), which is the donor of the A genome of polyploid wheat (Dvorak et al. 1988). Previous studies have shown that the chromosome 1A of bread wheat and 1A^m^ of *T. monococcum* recombine poorly in the presence of the *Pairing homeologous1* (*Ph1b*) gene, but that normal recombination can be restored through the use of the *ph1b* mutation (Dubcovsky et al. 1995). However, in the presence of the wild type *Ph1b* the reduction in recombination is not the same for all *T. monococcum* chromosomes, and some recombination was observed between the distal region of chromosomes 5A^m^ and 5A in a wild type hexaploid wheat background (Luo et al. 2000). This result agrees with the discovery of *T. monococcum* translocation of 14 different lengths in this study (Fig. 5), which suggests multiple 5A^m^ x 5A recombination events during the long breeding history of this introgression.

The ability of the *T. monococcum* chromosomes to recombine with the A-genome chromosomes (particularly in the *ph1b* background) has fueled the interest of breeders in the identification of resistance genes in this diploid species and its transfer to the commercial polyploid wheat species. Successful isolation and transfer of resistance genes from *T. monococcum* to hexaploid wheat include the stem rust resistance genes *Sr21* (Chen et al. 2015, 2018b), *Sr22* (Steuernagel et al. 2016), *Sr35* (Saintenac et al. 2013), *SrTm4* (Briggs et al. 2015), *Sr60* and *SrTm5* (Chen et al. 2018a, 2020); the leaf rust resistance genes *Lr63* (Kolmer et al. 2010) and *LrTM16* (Sodkiewicz et al. 2008) and the powdery mildew resistance genes *Pm1b* (Hsam et al. 1998), *Pm4d* (Schmolke et al. 2012) and *Pm25* (Shi et al. 1998).

Although *T. monococcum* shows good adult plant resistance against *Pst* (Chhuneja et al. 2008), only two stripe rust resistance QTLs, *QYrtm.pau-2A* and *QYrtb.pau-5A*, have been mapped from this species so far (Chhuneja et al. 2008). *QYrtb.pau-5A* was identified in *T. monococcum* subsp. *aegilopoides* accession pau5088 and was mapped on chromosome arm 5A^m^L flanked by simple sequence repeat (SSR) markers *barc151* and *cfd12*. Using the sequences of these two SSR markers, we determined the physical location of *QYrtb.pau-5A* in the reference genome of Arina*LrFor* was from 557.7 Mb to 561.9 Mb. Since *Yr34* was located distal to marker *pku5488F4R4* (700.7 Mb), we concluded that *QYrtb.pau-5A* and *Yr34* are likely two different genes.

Stripe rust resistance genes *Yr34* and *Yr48* were previously suggested to be the same gene on the basis of an allelism test and similar responses to different *Pst* races (Qureshi et al. 2018). Although the limited recombination within the *T. monococcum* 5A^m^L chromosome segment limits the value of the *Yr48* (L1) × *Yr34* (L2) allelism test, the absence of susceptible plants suggests that both *Yr48* and *Yr34* are located within the shorter 9.5 Mb segment (L2). This result supports (but does not prove) the suggestion that *Yr34* and *Yr48* are the same gene. Varieties Billings, Arina*LrFor* and SY Mattis carry the same L2 segment as WAWHT2046, suggesting that they also carry the *Yr34/Yr48* resistance gene. However, since the *Yr34/Yr48* causal gene has not been identified yet, we cannot rule out the possibility that these varieties carry a non-functional copy of this gene.

The presence of this alien *T. monococcum* translocation likely explains the segregation distortion and the suppression of recombination observed in the chromosome region carrying *Yr34* and *Yr48* (Lan et al. 2017; Lowe et al. 2011).

The regions of suppressed recombination were not identical for *Yr34* and *Yr48.* In the *Yr34* study (Qureshi et al. 2018), the authors reported recombination between *Yr34* and the awn inhibitor gene *B1* located at 698.5 Mb in CS RefSeq v1.0 (DeWitt et al. 2020). By contrast, the region of suppressed recombination for *Yr48* extended to *VRN2* at 698.2 Mb (Fig. 3b) (Lowe et al. 2011). This difference in recombination is supported in this study by the finding that the *T. monococcum* introgressions have different lengths in the donor of *Yr48* (L1 in PI 610750) and the donor of *Yr34* (L2 in WAWHT2046). The *B1* and *VRN2* loci are outside the translocation in WAWHT2046 and within the translocation in PI 610750.

### The 5A^m^L translocation has been used across a wide spatial and temporal range

We detected the 5A^m^L translocation in accessions from 50 countries, which suggests that it has been used in wheat breeding worldwide. However, the frequency of this translocation is not uniform across continents, ranging from less than 5% in South America, Africa and Oceania to 34.4% in Europe (Fig. S2 and Table S7). This data suggests that either the translocation has an older breeding history in Europe or it has been under stronger positive selection in Europe than in other regions. Although it is possible that the presence of stripe rust resistance gene *Yr34* contributed to the increased frequency of this segment, we cannot rule out the possibility that other favorable genes within this *T. monococcum* translocation favored its selection.

This wide geographic distribution of the *T. monococcum* introgression also suggests that it has a long history. Indeed, the screening of two large and diverse panels of wheat accessions with exome capture data (He et al. 2019; Pont et al. 2019) revealed the presence of the translocation in 11 wheat varieties released before 1931 (Table S5). Pedigree analysis of these varieties found that a wheat variety named LV-Mediterranean (or its derivatives) was frequent in the pedigrees of the varieties carrying the *T. monococcum* translocation. We confirmed the presence of the longest L1 translocation in Mediterranean accession CItr 5303 and the reduced L3 translocation in another Mediterranean accession (CItr 11587). It should be pointed out that these accessions were collected in different places of the USA nearly 100 years after its introduction in the US from Italy in 1819 under the name “Mediterranean” (Olmstead and Rhode 2002). Mediterranean was a very popular variety due to its better resistance to Hessian fly and rust than other varieties. Nearly 100 years after its introduction, Mediterranean occupied 2,770,000 acres and, in 1924, it was still grown on 600,000 acres (Ball 1930). Mediterranean’s long history and wide area of cultivation likely explain the heterogeneity of the Mediterranean samples maintained in the USDA-NSGC.

The comparison of the longest L1 introgression in Mediterranean and PI 610750 provides additional evidence of the ancestral origin of the *T. monococcum* segment in Mediterranean. In Mediterranean, we were not able to find the putative conversion events observed in PI 610750. The pedigree of PI 610750 suggests that the L1 segment from Mediterranean passed through at least 11 crosses to reach PI 610750. If we assume an average of three generations of self-pollination before fixation, this would imply that the L1 segment from PI 610750 passed though > 30 meiosis, providing multiple opportunities for conversion events. By contrast, if the L1 from Mediterranean CItr 5303 was never crossed and was never in heterozygous state, it had no opportunities for conversion events.

Taken together, these results suggest that this *T. monococcum* translocation may have provided *Pst* resistance for over 200 years and that it may represent one of the oldest alien introgressions in hexaploid wheat.

### Source of the* T. monococcum* introgression

Although we have established with high level of confidence that the introgressed segment originated from a cultivated *T. monococcum* subsp. *monococcum* and not from the related wild *T. monococcum* subsp. *aegilopoides*, we have not identified the exact accession of diploid wheat where this segment originated.

The closest *T. monococcum* subsp. *monococcum* accessions to the L1 segment are from Europe, which is consistent with the origin of Mediterranean in Italy. Two accessions from the UK are particularly close to the L1 introgression, pointing to the UK as a potential origin of the introgression. A more extensive survey of *T. monococcum* accessions and the sequencing of a larger number of loci will be necessary to provide a more conclusive answer to this question.

Most of the SNPs detected between L1 and L2 or Mediterranean are likely conversion events from *T. aestivum* chromosome 5A, since the same alleles were found in multiple hexaploid accessions. However, we found a 34-bp deletion linked to 4 SNPs that were not found in any of the sequenced genomes of *T. aestivum* but were detected instead in a group of four *T. monococcum* accessions from the Caucasus and Germany. If we assume that Mediterranean and PI 610750 have the same L1 translocation based on their shared proximal border (with a 0.2 Mb resolution), then the absence of these five polymorphisms in Mediterranean would indicate that they represent an introgression or conversion that occurred after the introgression of the *T. monococcum* segment in hexaploid wheat. We speculate that these polymorphisms may have originated from additional crosses with *T. monococcum.* Since wheat was grown extensively during the Roman Empire and after in an area that overlaps with *T. monococcum* geographical distribution, it would be interesting to investigate if this *T. monococcum* introgression has actually a much longer history.

### Candidate genes for *Yr34*

Most of the cloned disease resistance genes in wheat encode intracellular coiled-coil nucleotide-binding leucine-rich repeat (NLR) proteins (Chen et al. 2018a; Marchal et al. 2018; Saintenac et al. 2013; Wang et al. 2020a; Zhang et al. 2019, 2017), which recognize pathogen effectors and activate effector-triggered immunity (Jones and Dang 2006). However, we did not detect any typical NBS-NLR genes within the 9.5 Mb translocation in the genomes of Arina*LrFor* or SY Mattis, which suggests that *Yr34* likely belong to a different class of resistance genes.

In the L2 candidate region we identified six *RLKs*, four of which are expressed in wheat leaves infected with *Pst. RLKs* have been frequently associated with disease resistance in different plant species (Brueggeman et al. 2002; Hurni et al. 2015; Martin et al. 1993; Song et al. 1995; Wang et al. 1996). Two of the cloned stripe rust resistance genes, *Yr36* (Fu et al. 2009) and *Yr15* (Klymiuk et al. 2018) encode proteins with kinase domains, and similar to *Yr34*, provide broad spectrum resistance against *Pst* and have remained effective for many years. We have prioritized these *RLKs* for functional characterization to test if they are the causal genes for *Yr34*.

## Conclusions and practical implications

*Yr34* confers intermediate levels of resistance against virulent *Pst* races in a wide range of regions, including China (the current study), the United States (Lowe et al. 2011), Mexico (Lan et al. 2017), and Australia (Qureshi et al. 2018), indicating broad-spectrum resistance to different *Pst* races. In addition to its broad resistance, the fact that *Yr34* has remained effective for more than 100 years after its extensive deployment in commercial agriculture suggests that this gene provides durable resistance to *Pst*. However, since *Yr34* only provides partial resistance, it needs to be combined with other *Pst* resistance genes to confer economically useful levels of resistance.

The broad resistance spectrum and durability of *Yr34* makes this gene a desirable target for introgression into modern wheat varieties. For this purpose, we recommend the utilization of the shorter 9.5 Mb from Arina*LrFor*, WAWHT2046, Billings or SY Mattis. The L2 translocation provides similar levels of resistance as the L1 translocation, minimizes potential linkage drag, and reduces the chromosome area with limited recombination associated with the *T. monococcum* segment. Until *Yr34* is mapped more precisely, it is better to use a combination of a proximal marker (*pkuS5A7761F2R2*, currently a Sanger sequencing marker) and a distal (*pku5585F1R1*, Fig. 4) marker to confirm that the L2 segment was transferred without recombination. These CAPS and sequenced-based markers represent a useful tool to accelerate the deployment of this broad spectrum and durable resistance gene in modern wheat breeding programs.

## Supplementary Information

Below is the link to the electronic supplementary material.

Supplementary file1 (XLSX 725 kb)

Supplementary file1 (PDF 512 kb)
