# Supplementary material for: Stripe rust resistance gene Yr34 (synonym Yr48) is located within a distal translocation of Triticum monococcum chromosome 5AmL into common wheat
Source: Theor Appl Genet. 2021 Mar 31;134(7):2197–211. doi: 10.1007/s00122-021-03816-z (PMC8263425; doi:10.1007/s00122-021-03816-z)
Supplement: Supplementary file 2 — Supplementary file1 (PDF 512 kb) [file 122_2021_3816_MOESM2_ESM.pdf]

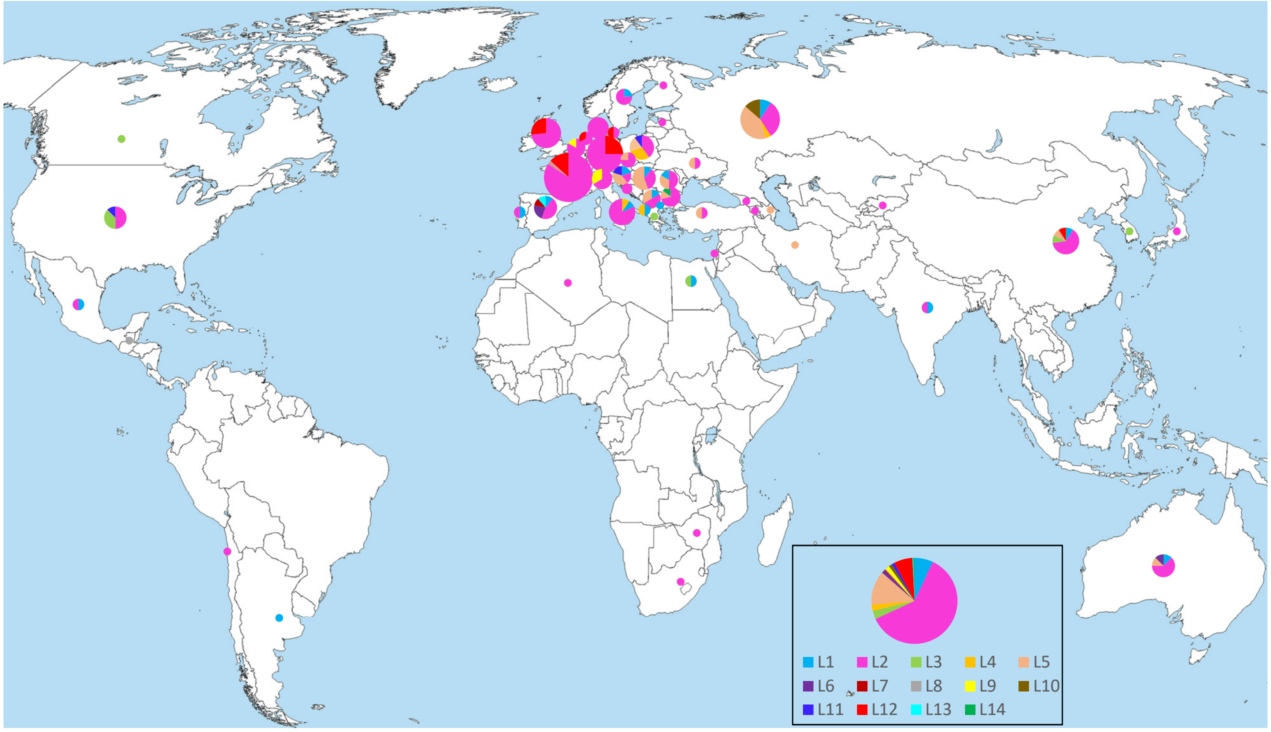

Fig. S1 Geographic distribution of 252 *T. aestivum* accessions carrying *T. monococcum* 5AL/5A^{m}Ltranslocations of 14 different lengths. 105 accessions were identified from the 1000 wheat exomes project (He et al. 2019) and 147 accessions from the 500 exomes project (Pont et al. 2019). Lines PI 610750, Billings, Arina*LrFor*, SY Mattis and WAWHT2046 characterized in this study are also included. The pie chart in the reference represents the proportion of accessions with each length class (L1 to L14). The smaller pie charts represent their relative distribution in different countries. Sky blue, L1 (18 accessions); Pink, L2 (157); Light green, L3 (8); Orange, L4 (6); Light pink, L5 (33); Purple, L6 (3); Crimson, L7 (1); Gray, L8 (1); Yellow, L9 (4); Brown, L10 (3); Blue, L11 (3); Red, L12 (18); Bright blue, L13 (1); and dark green, L14 (1).

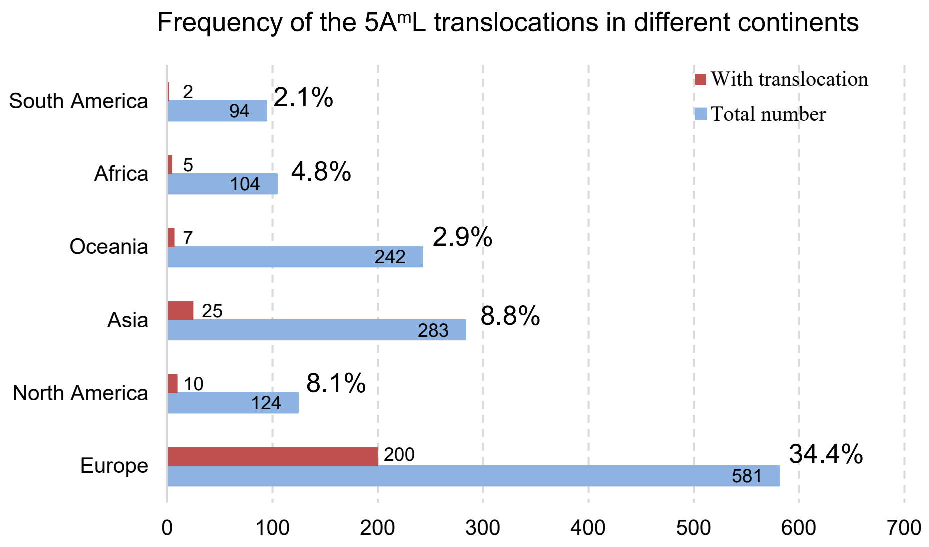

Fig. S2 Frequency of the $5A^{m}L$ translocation in different continents. A total of 252 wheat accessions carrying *T. monococcum*-wheat translocations were identified from 1,442 hexaploid wheat accessions. Accessions with “unknown” origin (3 with translocation out of 14 lines) were excluded in this figure.
